# Intersectional inequality in general and central obesity: cross-sectional UK Biobank study

**DOI:** 10.1038/s41366-025-01984-2

**Published:** 2026-01-05

**Authors:** Joseph Hutchinson, Natalie Darko, Rebecca Hardy, David Webb, Francesco Zaccardi, William Johnson

**Affiliations:** 1 School of Sport, Exercise and Health Sciences, Loughborough University, Loughborough, UK; 2 Department of Population Health Sciences, University of Leicester. Director of Inclusion, Leicester NIHR Biomedical Research Centre, Leicester General Hospital, Leicester, UK; 3 Diabetes Research Centre, University of Leicester. Consultant in Diabetes and Endocrinology, University Hospitals of Leicester NHS Trust, Leicester, UK; 4 Leicester Real World Evidence Unit, Diabetes Research Centre, University of Leicester, Leicester, UK

**Keywords:** Epidemiology, Risk factors, Obesity, Obesity, Epidemiology

## Abstract

**Background:**

People exist at a combination of different individual and neighbourhood deprivations. Each of these combinations may have a unique impact on health. However, little is known about the intersectional inequality of these combinations on general and central obesity, including when considering their demographics. This study aims to answer these questions.

**Methods:**

The sample comprised 452,339 participants from the UK Biobank study. Individuals were grouped into 320 intersectional strata according to their household income, neighbourhood deprivation, sex, ethnicity and age. Linear and logistic multilevel analysis of individual heterogeneity and discriminatory accuracy was used to establish the total, additive and interactive inequality of body mass index (BMI), fat mass index (FMI), and waist to height ratio (WHtR), as well as the associated obesity classifications.

**Results:**

6.5%, 25.2% and 9.1% of the total variation in BMI, FMI and WHtR, respectively, was due to inequality between the strata. Of this, 26.5%, 3.5% and 22.0% is interactive. 79, 58 and 93 strata for BMI, FMI and WHtR demonstrate a significant interactive effect. We found some patterns; for example, affluent white women have an advantaged interactive effect, whilst deprived black women have a disadvantaged effect. Meanwhile men experience the inverse relationship. The relationship between individual and neighbourhood deprivation is not universally experienced by all strata. For example, black men living in areas of high deprivation have higher BMIs as their household income increases.

**Conclusions:**

A large proportion of variation in general and central obesity is due to intersectional inequality, with up to 26.5% being interactive. It is important that these intersectional effects are considered when designing policy interventions to avoid policy failure, such as by focussing on groups with high total and interactive risk.

## Introduction

The worldwide prevalence of obesity has increased over recent decades [1]. In the United Kingdom, the proportion of adults with obesity increased from 14.9% in 1993 to 28.0% in 2019 [2]. The cause of obesity is a complex interaction of factors including, but not limited to, individual factors (such as education) and the social environment (such as socioeconomic deprivation) [3].

In high-income countries, the most socially disadvantaged groups have the greatest rates of obesity [4]. Deprivation can occur at the individual or neighbourhood level. Deprivation at the individual and neighbourhood levels separately impact obesity, in general both having a positive association [5]. However, individual deprivation may be modified by neighbourhood deprivation or vice-versa. For example, as individual deprivation changes, the impact of neighbourhood deprivation may change. This modification may be synergistic or antagonistic. However, this association between deprivation and obesity varies by ethnicity, age and sex [5, 6]. For example, women experience a stronger effect of deprivation on obesity than men [6]. In real life, an individual exists at a social position comprised of their unique combination of individual and neighbourhood deprivation, age, sex and ethnicity. As such, the social position is a complex interaction of the impact from these factors. Unfortunately, the general associations oversimplify this relationship and will likely fail to accurately consider this complex interaction. Therefore, the unique risk for individual social positions may be mis-estimated without considering these factors interactively.

Intersectionality is a critical theoretical framework that examines how systems of power (e.g. racism or sexism) intersect to shape individual and group health outcomes [7, 8]. As originally articulated by Kimberlé Crenshaw [7, 8], and further developed in public health by scholars like Hankivsky [9], intersectionality includes attention to how structural inequities determine risk. This paper theorises that these factors interact to create a unique social position where elements of privilege and disadvantage combine to impact obesity risk [10–12]. Thus, we consider that an individual’s risk consists of the additive effect of their social characteristics as well as the interactive effect of these factors. This contrasts with the purely additive approach to inequality, where the factors comprising their social position are only considered cumulatively [13]. For example, when we apply an intersectional approach, we may find the combination of low individual and neighbourhood deprivation results in lower obesity risk than expected by the addition of these individual factors. This would indicate that an advantaged interactive effect is experienced in that context [14]. It is important that interactive effects are modelled, as an oversimplistic additive approach may mis-estimate risks for certain groups. This may provide opportunities to improve policy, such as better targeting resources.

A novel way of analysing this intersectionality is Multilevel Analysis of Individual Heterogeneity and Discriminatory Accuracy (MAIHDA), which has several advantages over pre-existing techniques [14].

Body mass index (BMI) is most frequently used to define obesity, however it is criticised for being misrepresentative as it incorporates both lean and fat mass [15]. Therefore, individuals at extremes of lean mass will have inaccurate estimations of obesity when using BMI. This may vary by demographic and deprivation characteristics. Fat mass index (FMI) is similar to BMI but does not include lean mass [16, 17]. As such, it may be a better measure of obesity. The anthropomorphic distribution of fat is also important, as even people with a normal weight have increased cardiovascular disease, diabetes mellitus and total mortality if their fat is distributed centrally. Therefore waist to height ratio (WHtR) is an important additional measure [18, 19]. As such, we will model all these variables as well as their related obesity classifications.

This study aims to use I-MAIHDA to analyse the combined effects of ethnicity, sex, age, income, and neighbourhood deprivation on general and central obesity, distinguishing between additive and interactive components of intersectional inequality.

## Methods

### UK Biobank

UK Biobank is a major biomedical database of approximately 500,000 participants located in England, Scotland and Wales [20]. The baseline data collection ran from 2006-2010. Participants completed a touchscreen questionnaire, underwent a range of physical measures and provided biological samples.

### Deprivation and demographics

Ethnicity in UK Biobank was self-reported using Office for National Statistics categories [21]. Self-reporting may be influenced by culture, heritage or physical appearance [21]. For our analysis four groupings were defined, specifically: White (British, Irish, Any other White background), mixed (White and Black Caribbean, White and Black African, White and Asian, any other Mixed background), Asian (Indian, Pakistani, Bangladeshi, Chinese, any other Asian background), and Black (Caribbean, African, any other Black background) [22].

Average total household income before tax was used as our measure of individual socioeconomic position [23]. Participants selected an income category and were able to select yearly, weekly or monthly equivalents. Participants were grouped as <£18,000, £18,000 to £30,999, £31,000 to £51,999, and ≥52,000 per year. 64,269 respondents preferred not to say or did not know their household income and were included as a further category.

Index of Multiple Deprivation (IMD) was used as our measure of neighbourhood deprivation [24, 25]. IMD is a composite measure of area level deprivation combining measures of housing, crime, education, employment, health, income and the living and physical environment. England, Scotland and Wales combine different measures to create different scores. Unfortunately, Abel et al.’s technique of adjusting the IMD scores to enable the combination is not possible in the UK Biobank dataset [26]. Nevertheless, the English and Scottish IMD are comparable [26, 27]. Therefore, the Welsh observations were excluded from the analysis. The participants were then grouped into low, moderate-low, moderate-high, and high deprivation neighbourhoods by applying the quartile cut offs from the respective whole population datasets [28, 29].

Age at recruitment was categorised into <55 and aged 55 or above [30]. Sex was kept as female or male [31]. This created 320 strata (required for MAIHDA [described below]), from the combination of:

- Household Income (Individual Deprivation): <£18,000, £18,000-£31,000, £31,000-£52,000, >£52,000 and prefer not to say/don’t know;
- Index of Multiple Deprivation (Neighbourhood Deprivation): high, moderate-high, moderate-low neighbourhood and low;
- Sex: male or female;
- Ethnicity: White, Black, Asian, Mixed;
- Age: <55, ≥55 years.

### Outcomes

BMI and FMI were used as measures of general obesity while WHtR was our measure of central obesity [32]. Weight, height, body fat and waist circumference were measured with WHtR, BMI and FMI calculated [33] There were some biologically implausible values that were set as missing, as per previous research (BMI < 14 kg/m^2^ [n = 12] and >70 kg/m^2^ [n = 1] and waist circumference (≤51 cm [n = 8] and ≥190 cm [n = 1]) [34, 35]. BMI, FMI and WHtR were positively skewed so they were log transformed using y=100log_e_(x): this creates sympercents (s%) for the model outcomes which are symmetric percentage differences [36].

Obesity classifications were applied according to national guidelines, specifically; normal weight (<25 kg/m^2^), overweight (25-29.9) and obesity (≥30)for BMI and normal (0.5), increased (≥0.5 and <0.6) and high (≥0.6) for WHtR [37]. Sex-specific FMI obesity classifications were applied. Females were classified as normal weight (<9 kg/m2), overfat (9-12.9 kg/m2) and obesity (≥13 kg/m2) [16] and males as normal weight (<6 kg/m2), overfat (6-8.9 kg/m2) and obesity ( ≥ 9 kg/m2) [16]. Underweight individuals (n = 2551) were included in the normal weight categorisation.

### MAIHDA

Most studies analysing intersectional inequality use single-level regression [38], which has theoretical and methodological limitations. Evans et al. popularised intersectional multilevel analysis as a novel approach to modelling intersectionality [39]. The acronym MAIHDA was introduced by Merlo in an accompanying commentary [40]. That commentary situated intersectional MAIHDA as one application of a broader variance-based framework, originally developed for geographical and institutional epidemiology [40]. Intersectional applications typically show larger variance partition coefficients (VPCs) than geographical studies and therefore gained more visibility. Nevertheless, MAIHDA should be understood as a general framework for contextual epidemiology, applicable across multiple settings. I-MAIHDA considers how every individual is nested within a unique social strata, such as a categorised combination of sex, ethnicity, age, and individual and neighbourhood deprivation. It then allows the analysis of general contextual effects (proportion of variance in outcome explained by the strata structure, as opposed to between individuals), specific contextual effects (strata-specific effect, which is how much an individual stratum differs from the grand mean) and the discriminatory accuracy (accuracy of strata membership to classify an outcome) [41]. It therefore enables a better understanding of how social context impacts risk of the outcome. Statistically, MAIHDA has several benefits over a fixed-effect model with interactions, usually used to model intersectionality. Shrinkage of the random-effects makes it more reliable for analysing strata with smaller sample sizes [14, 42]. Similarly, in a fixed-effect approach additional variables increase interactions geometrically, making analysis challenging [14]. MAIHDA also uses variance partitioning to analyse separate additive and interactive effects. Additive inequality describes the sum of the component strata variables, whilst interactive inequality describes the unique way they interact. Intersectional inequality comprises both the additive and interactive components.

The procedure of I-MAIHDA is detailed in full by Evans et al. [14]. Two multilevel models are fit for each outcome:

- Model 1 - a “null” random intercept model with the individuals (level 1) nested within their unique strata (level 2). This includes no fixed-effects and we estimate the general contextual effects (VPC) which is the stratum-level variance divided by the total variance (${VPC}=\,\frac{{\sigma }_{\mu }^{2}}{{\sigma }_{\mu }^{2}+\,{\sigma }_{\epsilon }^{2}}$))) and specific contextual effects (stratum-level predictions).
- Model 2: - a “main-effects” random intercept model with the variables used to define the strata added as level 2 fixed-effects. The VPC is then calculated along with the proportional change in variance (PCV) which measures the proportion of the stratum effect that is additive, with 1-PCV measuring the proportion that is interactive. PCV is calculated as the difference between the “null” and “main-effects” models stratum-level variance divided by the “null” stratum level variance (${PCV}=\frac{{\sigma }_{\mu \left({null}\right)}^{2}{-\sigma }_{\mu ({main})}^{2}}{{\sigma }_{\mu \,\left({null}\right)}^{2}}$).

Interpretation of the results should be considered a descriptive exploration of how adiposity varies between intersectional strata as opposed to what has caused this variation to arise.

### Data analysis

Complete case analysis was performed as only 5.9% of all observations had 1 or more missing variables. Descriptive statistics were then reported.

We aimed for our set of strata to be as diverse as possible. MAIHDA shrinks strata estimates to the overall mean inverse to the strata sample size, which improves reliability in groups with small sample sizes [14]. Simulation studies suggest robustness to a minimum of 10 participants per strata [14, 43, 44]. However, due to the scientific importance of studying under-represented groups some degree of imprecision is tolerable. Nevertheless, we wanted results to be reliable for all strata. Therefore, strata development was a balance between representing marginalised groups and statistical limitations. First all possible combinations of interactions were developed from the constituent variables. Sparse categories (<10 participants) were then collapsed, such as Indian into Asian or >£100,000 household income into >£52,000.

General linear MAIHDA models were developed for each of the continuous variables—BMI, FMI and WHtR. For each of the obesity classifications (BMI, FMI and WHtR) binary logistic MAIHDA models were used. Separate models for comparing overweight to normal weight and obesity to normal weight (or equivalent) were developed. Effectively, this constructs a multilevel multinomial logistic model which is not directly supported in R.

It is not possible to directly estimate level 1 variance in logistic models. A latent variable approach was used with level 1 variance set at 3.29 [14]. Separate predictions for each strata were estimated. First, predictions were made using the fixed-effects, which is the additive inequality. Second, predictions were made using the fixed and random-effects, which is the additive and interactive effects. The gap between these predictions is the difference from the interactive effect. These predictions were tabulated. Point estimates of the full predictions were plotted and the interactive effects were presented in a heatmap.

All analyses were done in R version 4.4.1. The study was conducted and reported following STROBE guidelines, with the checklist available in the supplement.

### Ethical approval and consent to participate

All methods were performed in accordance with the relevant guidelines and regulations. The study was conducted under the UK Biobank ethical approval by the North West Multi-centre Research Ethics Committee. All participants of the UK Biobank has provided informed consent.

## Results

Descriptive statistics are detailed in Tables 1 and S2. There were 320 strata; 3 strata had <10 participants, specifically:

- Male, <£18,000, Black, <55 and low neighbourhood deprivation (n = 6);
- Male, prefer not to say/don’t know, Black, 55 or above, low neighbourhood deprivation (n = 9);
- Male, £18,000-£31,000, Black, <55, low neighbourhood deprivation (n = 9).
- These strata will be more conservatively estimated.

**Table 1:** Descriptive statistics.

Characteristic	Female N = 246,639	Male N = 204,700
Ethnicity		
Asian	5085 (2.1%)	5205 (2.5%)
Black	4201 (1.7%)	3052 (1.5%)
Mixed	3964 (1.6%)	2686 (1.3%)
White	233,389 (95%)	193,757 (95%)
Age category (years)		
<55	97,031 (39%)	77,063 (38%)
55 or above	149,608 (61%)	127,637 (62%)
Household Income (Individual Deprivation)		
>£52000	48,165 (20%)	52,167 (25%)
£31,000–£52,000	51,963 (21%)	49,120 (24%)
£18,000–£31,000	53,803 (22%)	44,698 (22%)
<£18,000	50,196 (20%)	37,085 (18%)
Prefer not to say/Don’t Know	42,512 (17%)	21,630 (11%)
Neighbourhood deprivation		
Low neighbourhood deprivation	85,395 (35%)	69,511 (34%)
Moderate—low neighbourhood deprivation	70,602 (29%)	57,347 (28%)
Moderate—high neighbourhood deprivation	50,931 (21%)	41,917 (20%)
High neighbourhood deprivation	39,711 (16%)	35,925 (18%)
General obesity—fat mass index		
Fat mass index	10.19 (3.78)	7.22 (2.66)
Normal	106,055 (43%)	70,027 (34%)
Overweight	92,222 (37%)	91,992 (45%)
Obesity	48,362 (20%)	42,681 (21%)
General obesity—body mass index		
Body mass index	27.05 (5.15)	27.81 (4.20)
Normal weight	98,345 (40%)	51,681 (25%)
Overweight	90,763 (37%)	101,445 (50%)
Obesity	57,531 (23%)	51,574 (25%)
Central obesity—waist to height ratio		
Waist to height ratio	0.52 (0.08)	0.55 (0.06)
Normal	110,445 (45%)	42,355 (21%)
Increased	96,856 (39%)	120,888 (59%)
High	39,338 (16%)	41,457 (20%)

Categorical variables are shown as number (percentage). Continuous variable are shown as mean (SD).

### Body mass index

Descriptive statistics are detailed in Table 1. 23% of females and 25% of males had obesity according to BMI. Variation partition statistics are detailed in Table 2. In the ‘null’ model 6.48% and 11.08% of variation in BMI and obesity classification, respectively, was between strata, due to inequality defined by our strata. 73.46% and 80.49% of this between effect was additive respectively, with 26.54% and 19.51% being interactive.

**Table 2:** Model variance by individual (level 1) and strata (level 2) effects.

		Individual variance	Strata variance	Variance partition coefficient (%)	Proportional change in variance (%)	1 – Proportional change in variance (%)
Body mass index (BMI)—log scale	Model 1	264.17	18.31	6.48	–	–
	Model 2	264.17	4.86	1.81	73.46	26.54
Fat mass index (FMI)—log scale	Model 1	1331.30	448.89	25.22	–	–
	Model 2	1331.36	15.54	1.15	96.54	3.46
Waist to height ratio—log scale	Model 1	168.89	16.84	9.07	–	
	Model 2	168.89	3.71	2.14	77.97	22.03
Overweight v normal weight (BMI)	Model 1	3.29	0.19	5.46	–	–
	Model 2	3.29	0.03	0.90	84.21	15.79
Obesity v normal weight (BMI)	Model 1	3.29	0.41	11.08	–	–
	Model 2	3.29	0.08	2.37	80.49	19.51
Overweight v normal weight (FMI)	Model 1	3.29	0.14	4.08	–	–
	Model 2	3.29	0.01	0.30	92.65	7.35
Obesity v normal weight (FMI)	Model 1	3.29	0.36	9.86	–	–
	Model 2	3.29	0.06	1.79	83.33	16.67
Central obesity (Increased v Healthy WHtR)	Model 1	3.29	0.44	11.80	–	–
	Model 2	3.29	0.03	0.90	93.18	6.82
Central obesity (High v Healthy WHtR)	Model 1	3.29	0.69	17.34	–	–
	Model 2	3.29	0.11	3.24	81.79	18.21

BMI, FMI and WHtR are log transformed. The classification outcomes level 1 (individual) variance is set at 3.29, using the latent variable approach for logistic models. Variance partition coefficient (VPC) details the proportion of variance at level 2, with model 1 VPC describing the inequality by intersectional strata. Similarly, 1-proportional change in variance details the proportion of the strata effect remaining after accounting for additive effects, which can be considered the interactive effect.

Fixed-effect estimates are detailed in Tables 3 and 4. Male sex (symmetrical % (s%) 1.03 (95% Confidence interval (CI): 0.45, 1.62)), age 55 or above (1.37 s% (0.78, 1.96)), <£18,000 household income (3.08 s% (2.14, 4.02)), Black ethnicity (5.49 s% (4.64, 6.33)) and high neighbourhood deprivation (4.56 s% (3.71, 5.41)) had higher BMI relative to female, age <55, >£52,000, white ethnicity and low neighbourhood deprivation respectively. Asian ethnicity had 3.01 s% (2.24, 3.78) lower BMI than white ethnicity. These effects were consistent with the BMI obesity classification.

**Table 3:** Fixed-effect estimates for the main-effects models of the continuous variables; body mass index, fat mass index and waist to height ratio.

	Body mass index—Sympercent (95% CI)	Fat mass index—Sympercent (95% CI)	Waist to height ratio—Sympercent (95% CI)
Intercept	25.77 (25.51, 26.03)	8.58 (8.42, 8.74)	0.48 (0.48, 0.49)
Sex (Female reference)			
Male Sex	1.03 (0.45, 1.62)	−37.93 (−39.05, −36.82)	3.56 (3.05, 4.06)
Age (<55 Reference)			
Age 55 or above	1.37 (0.78, 1.96)	8.05 (6.93, 9.17)	4.15 (3.65, 4.66)
Household income (>£52000 Reference)			
£31,000–£52,000	1.43 (0.49, 2.38)	2.70 (0.91, 4.50)	1.73 (0.92, 2.53)
£18,000–£31,000	1.60 (0.66, 2.54)	3.09 (1.30, 4.88)	2.54 (1.73, 3.34)
<£18,000	3.08 (2.14, 4.02)	5.86 (4.07, 7.65)	4.44 (3.64, 5.25)
Prefer not to say/Don’t know	2.28 (1.34, 3.21)	4.52 (2.74, 6.30)	3.55 (2.75, 4.35)
Ethnicity (White reference)			
Asian	−3.01 (−3.78, −2.24)	−1.47 (−2.92, −0.02)	2.27 (1.61, 2.93)
Black	5.49 (4.64, 6.33)	10.22 (8.60, 11.84)	2.75 (2.03, 3.47)
Mixed	−0.07 (−0.88, 0.74)	−0.32 (−1.88, 1.23)	1.20 (0.50, 1.89)
Neighbourhood deprivation (Low deprivation reference)			
Moderate—Low	1.58 (0.72, 2.45)	3.38 (1.73, 5.03)	1.33 (0.59, 2.06)
Moderate—High	2.85 (2.01, 3.70)	6.00 (4.39, 7.61)	2.56 (1.84, 3.29)
High	4.56 (3.71, 5.41)	9.48 (7.85, 11.10)	3.97 (3.24, 4.70)

The estimates are sympercents which are symmetrical percentage differences. These are relative to the reference group (female sex, income <£18,000, white ethnicity, aged < 55 and in a low deprivation neighbourhood).

**Table 4:** Fixed-effect estimates for the main-effects models of the obesity classification variables.

	Overweight v Normal body mass index	Obesity v Normal body mass index	Overweight v Normal fat mass index	Obesity v Normal fat mass index	Increased v Normal waist to height ratio	High v Normal waist to height ratio
	Odds ratio (95% CI)	Odds ratio (95% CI)	Odds ratio (95% CI)	Odds ratio (95% CI)	Odds ratio (95% CI)	Odds ratio (95% CI)
Intercept	0.68 (0.63, 0.74)	0.35 (0.31, 0.40)	0.53 (0.50, 0.57)	0.21 (0.19, 0.24)	0.47 (0.43, 0.51)	0.10 (0.09, 0.12)
Sex (Female reference)						
Male Sex	1.85 (1.75, 1.95)	1.25 (1.15, 1.36)	1.43 (1.37, 1.49)	1.04 (0.97, 1.12)	2.75 (2.59, 2.92)	1.92 (1.74, 2.11)
Age (<55 Reference)						
Age 55 or above	1.28 (1.21, 1.35)	1.23 (1.13, 1.33)	1.53 (1.47, 1.59)	1.64 (1.52, 1.76)	1.80 (1.70, 1.91)	2.40 (2.19, 2.64)
Household income (>£52,000 Reference)						
£31,000–£52,000	1.14 (1.05, 1.25)	1.24 (1.09, 1.41)	1.14 (1.07, 1.22)	1.24 (1.10, 1.40)	1.19 (1.09, 1.30)	1.41 (1.21, 1.64)
£18,000–£31,000	1.08 (0.99, 1.17)	1.24 (1.09, 1.41)	1.17 (1.10, 1.25)	1.32 (1.18, 1.49)	1.29 (1.18, 1.42)	1.74 (1.50, 2.02)
<£18,000	1.14 (1.05, 1.25)	1.60 (1.41, 1.82)	1.23 (1.16, 1.32)	1.72 (1.52, 1.93)	1.45 (1.32, 1.59)	2.57 (2.21, 2.98)
Prefer not to say/Don’t know	1.14 (1.05, 1.24)	1.44 (1.27, 1.64)	1.20 (1.13, 1.28)	1.53 (1.36, 1.73)	1.39 (1.27, 1.52)	2.09 (1.80, 2.42)
Ethnicity (White reference)						
Asian	0.89 (0.83, 0.95)	0.62 (0.55, 0.68)	1.14 (1.07, 1.20)	0.82 (0.74, 0.91)	1.60 (1.48, 1.73)	1.56 (1.38, 1.77)
Black	1.68 (1.54, 1.84)	2.27 (2.01, 2.56)	1.57 (1.46, 1.69)	1.97 (1.77, 2.20)	1.50 (1.37, 1.64)	1.60 (1.39, 1.84)
Mixed	1.04 (0.96, 1.12)	1.00 (0.89, 1.11)	1.04 (0.97, 1.11)	0.99 (0.89, 1.10)	1.20 (1.10, 1.30)	1.23 (1.08, 1.41)
Neighbourhood deprivation (Low deprivation reference)						
Moderate—Low	1.13 (1.04, 1.22)	1.27 (1.13, 1.43)	1.13 (1.06, 1.20)	1.30 (1.16, 1.45)	1.13 (1.04, 1.23)	1.37 (1.19, 1.57)
Moderate—High	1.20 (1.11, 1.30)	1.57 (1.40, 1.76)	1.21 (1.14, 1.29)	1.59 (1.43, 1.77)	1.27 (1.17, 1.38)	1.80 (1.57, 2.06)
High	1.27 (1.18, 1.38)	1.99 (1.77, 2.24)	1.30 (1.23, 1.38)	2.03 (1.82, 2.26)	1.39 (1.28, 1.52)	2.33 (2.03, 2.66)

These are odds ratios for being in the overweight/obesity category compared to the normal category, relative to the reference group (female sex, income <£18,000, white ethnicity, aged *<* 55 and in a low deprivation neighbourhood).

**Fig. 1:**
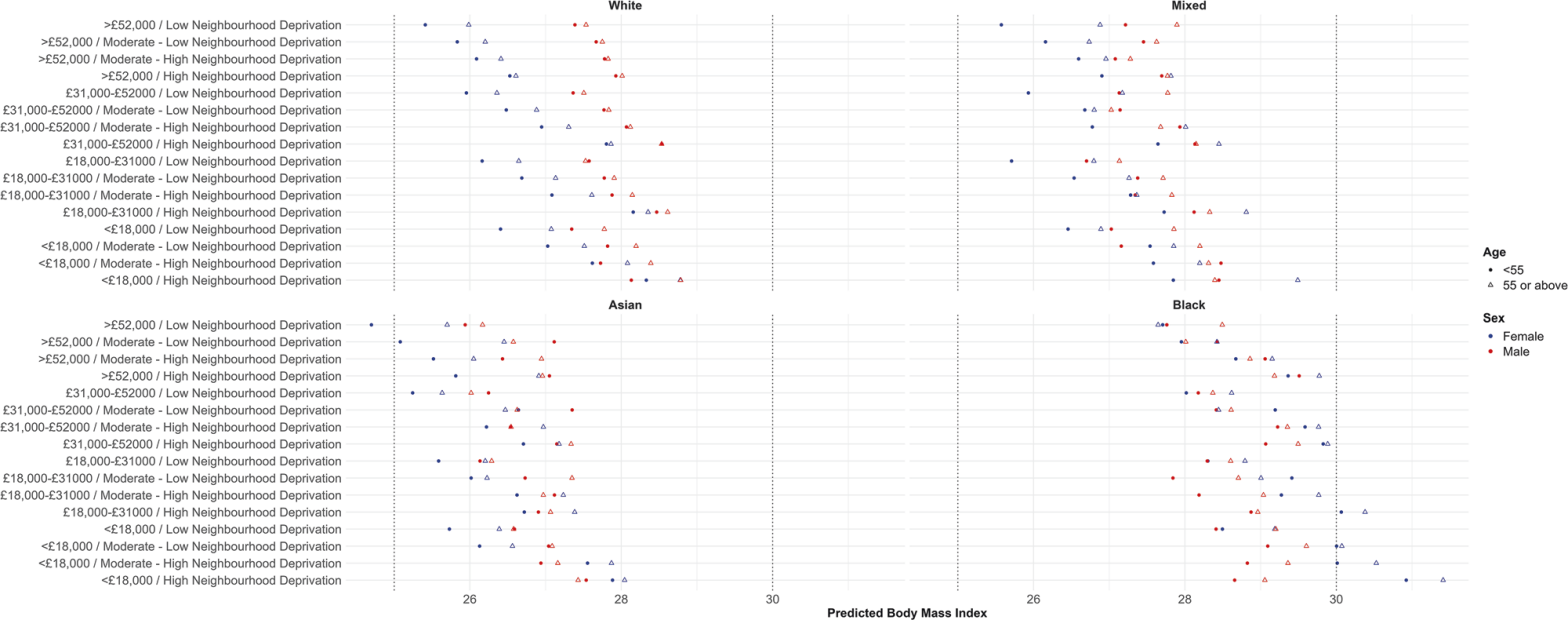
Predicted body mass index for the individual strata by ethnicity groupings. Predictions are made used fixed and random-effects, so represent both additive and interactive effects. Household income and neighbourhood deprivation groupings are detailed on the y axis. Male observations are in red, with female observations in blue. Aged *<* 55 is plotted with a circle, aged 55 or above is a triangle. Dotted vertical lines indicate the overweight (BMI 25 kg/m^2^) and obesity (BMI 30 kg/m^2^) classification thresholds.

Predicted BMI and obesity classifications are detailed in Tables S3, S5 and S6 and Fig. 1. Black women living in high deprivation neighbourhoods with low income had the highest predicted BMI and obesity classification. Generally, younger high income Asian and White women living in low deprivation neighbourhoods had the lowest predicted BMI and obesity classifications. This relationship was observed for men, but is less extreme.

**Fig. 2:**
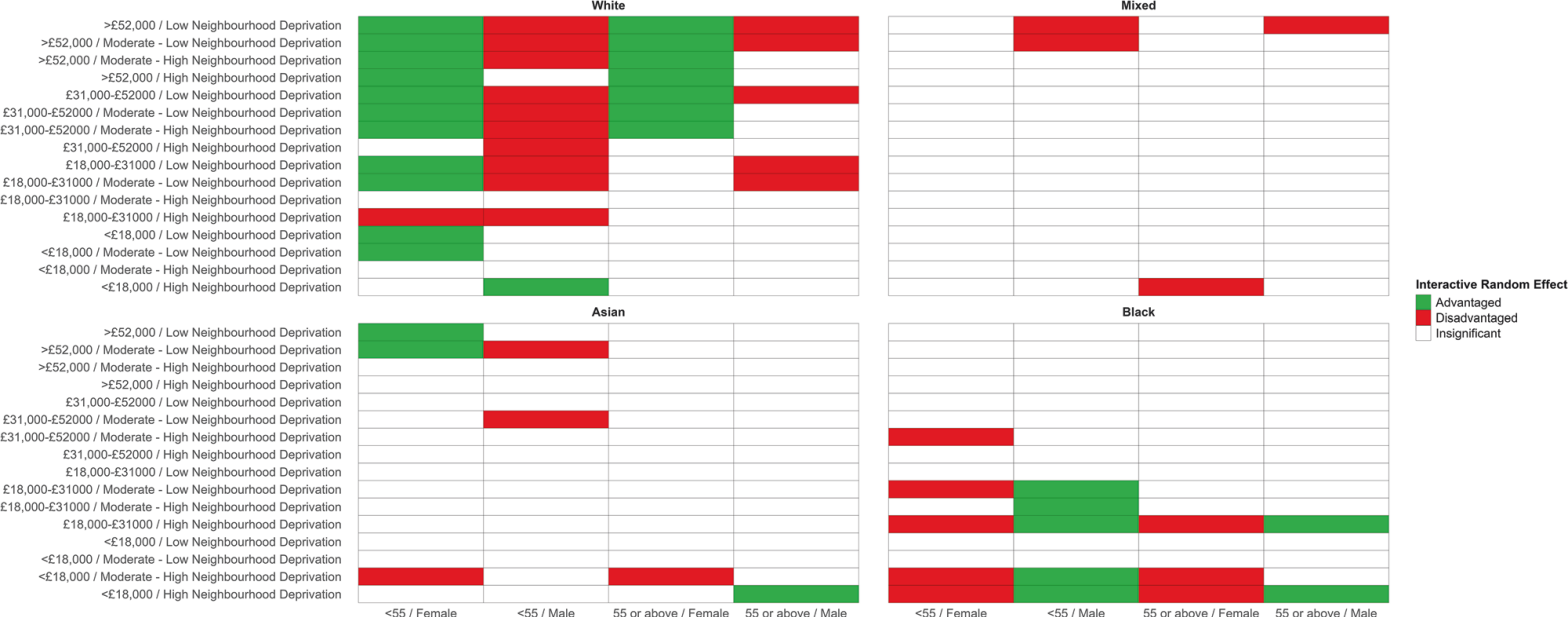
Heatmap of interactive random-effects per strata for body mass index, by ethnicity grouping. Statistically significant disadvantaged interactive effects are red, statistically significant advantaged interactive effects are green. Statistically insignificant interactive effects are white.

There is a sex difference in the interactive effect experienced between Black and White men and women (Fig. 2), where most of the significant interactive effects are observed. Generally deprived black women experience an interactive effect that increases their BMI whilst affluent white women experience an interactive effect that decreases their BMI. However, this is inverse for men. As such, for males the interactive effect acts to narrow the disparity between Black and White individuals, whilst for females it widens the disparity. For example, the BMI gap for doubly affluent women aged <55 increased from 1.48 to 2.21, whilst it decreased from 1.49 to 0.40 for the otherwise same men.

White males aged <55 with the lowest individual and neighbourhood deprivation have a predicted BMI based on fixed and random-effects of 26.4 and 27.4 respectively, with a significant interactive effect of 3.7% (2.6, 4.9). Meanwhile, White women in the otherwise same social strata have 26.1, 25.4 and -2.8% (-3.8, -1.7) respectively. This means the difference in BMI increases from 0.3 to 2.0 when considering interactive effects. Meanwhile, the range of BMI increased from 4.9 kg/m^2^ to 6.7 kg/m^2^ when considering the interactive effect in addition to the additive effect, an increase of 1.8 kg/m^2^ (26.9%).

The effect of individual deprivation is reduced in low deprivation neighbourhoods, whilst the impact of neighbourhood deprivation is lower for high-income individuals. For example, White women aged <55 with affluent individual deprivation have a BMI gap of 0.12 between the lowest and highest deprivation neighbourhoods. Whilst <£18,000 has a gap of 1.97. However, certain groups experience other associations; for example, for Black men living in areas of higher deprivation a higher income is associated with a higher predicted BMI.

### Fat mass index

20% of females and 21% of males had obesity according to FMI (Table 1). 25.2% and 9.9% of variation in FMI and obesity classification was between strata, due to inequality defined by our strata (Table 2). 96.5% and 83.3% of this between effect was additive respectively, with 3.5% and 16.7% being interactive. As such, there is much more inequality when considering FMI as opposed to BMI, yet less of this inequality is interactive.

As expected, male sex had a 37.9 s% (36.8, 39.1) lower FMI than female sex (Table 3). When compared to BMI the direction of the fixed-effects were consistent. However, the magnitude is greater with FMI, for example high neighbourhood deprivation has 9.48 s% (7.85-11.10) higher FMI but only 4.56 s% (3.71-5.41) higher BMI, relative to low neighbourhood deprivation.

Predicted and interactive effects for FMI had similar associations as with BMI (Tables S4, S14, S15 and Figs. S1 and S3). However, with FMI a consistent disadvantaged interactive effect was observed for lower income White men aged >55 which was not seen with either BMI or WHtR.

### Central obesity (waist to height ratio)

16% of females and 20% of males had high WHtR (Table 1). 9.1% and 17.3% of variation in WHtR and obesity classification was between strata, due to inequality defined by our strata (Table 2). 78.0% and 81.8% of this between effect was additive respectively, with 12.0% and 18.2% being interactive.

Males sex was associated with a 3.56 s% (3.05, 4.06) higher WHtR relative to females. Asian and Black ethnicities were associated with a 2.27 s% (1.61, 2.93) ad 2.75 s%(2.03, 3.47) higher WHtR than white ethnicity respectively.

The interactive effects were similar to that observed with BMI, however a greater number of the strata have significant effects within the white ethnicity. Deprived Asian men aged ≥55 had a large probability of increased or high WHtR up to 92.75%, whilst affluent White women aged <55 had much lower probability, as low as 34.19%; representing a range of 58.56%.

## Discussion

### Summary

The inequality of obesity is more complex than when analysed from an additive perspective. 11.1% and 17.3% of variation in general and central obesity is due to our strata, of which 19.5% and 18.2% is interactive. When considering the interactive effect the range of BMI increases by 1.8 kg/m^2^ (26.9%). These interactive effects are complex. Black and White participants most frequently had a significant interactive effect. In general, high-income White women experienced an advantaged interactive effect, while socioeconomically deprived Black women experienced a compounded disadvantage. This pattern widens ethnic disparities in obesity among women. However, for men, the relationship was generally inverse.

### Comparison with existing literature

This study found that both individual and neighbourhood deprivation are associated with increased general and central obesity; congruent with previous literature for high-income countries [4, 5, 45, 46]. However, in the most affluent individual or neighbourhood groups the impact of the alternate measure was attenuated. This suggests that there is a protective effect of affluency.

The positive association with deprivation was not observed by all strata. For example, for males of Black ethnicity living in areas of high deprivation, higher household income was associated with higher BMI. This has potential policy implications, as focussing on lower income individuals may miss people most at risk.

The interactive effects vary across social strata, leading to different interpretations. For example, among women, the interactive effect contributed to a widening of ethnic disparities between White and Black groups, whereas among men, it narrowed them. The greatest total inequality is observed for FMI. Therefore, using FMI in clinical practice may better represent health inequalities. The models comparing overweight to normal weight had lower between-effect than for obesity, suggesting inequality is more important for obesity.

For BMI, which is not sex-specific, 6.48% of variation is due to inequality, of which 26.5% is interactive. This is congruent with previous research which identified interactive effects of 13.7% and 35.3% [39, 47].

This study extends previous studies examining intersectionality of obesity using MAIHDA [39, 47, 48]. Holman et al. studied how individual and neighbourhood deprivation, impacts biomarkers of ageing within UK biobank [48]. One of these biomarkers of ageing was waist circumference. Our study uses multiple measures of general and central obesity. Further, replicating their strata structure resulted in empty strata, potentially mis-estimating random-effects. Evans et al. and Hernandez-Yumar have also used MAIHDA to analyse BMI [39, 47]. These studies did not consider neighbourhood deprivation, were conducted outside of the UK, had smaller sample sizes (32,788 and 14,190) and either a simpler strata structure (108) or many strata (10%) with <10 observations. Therefore, this study should provide a more accurate and detailed understanding of obesity.

### Implications

This study highlighted how the impact of deprivation on obesity is more complex than when understood from an additive perspective. This can result in associations for individual strata that are inverse to that of previous literature. This is theoretically rooted in intersectionality where our findings suggest there is a large unique effect from an individual’s intersectional social strata on their risk of obesity [7, 8]. Classically these effects are considered to emerge from systems of power; however there may be additional mechanisms for these effects (e.g. food environment, culture or healthcare) which may provide opportunities for public health interventions.

This highlights the importance of ensuring intersectionality is modelled when analysing inequality. If the unique experiences of certain social groups are not considered, then estimated group risks will be wrong. For example, in this study we would have underestimated the obesity risk for deprived Black women. In effect, this results in a 1.8 kg/m^2^ (26.9%) underestimation in the range of BMI. Similarly, MAIHDA enables the construction of many strata which results in a more detailed understanding.

Caution is required when interpreting predicted outcomes and intersectional effects. For example, whilst some Black men experience an advantaged interactive effect, they still have high predicted BMIs. It is critical that this is interpreted accurately to avoid poorly designed policy.

This study demonstrates how MAIHDA can be used to identify groups at particular risk of health inequalities. In this case, deprived black women living in areas of high deprivation both have high probability of overweight and obesity (87.32%) and a disadvantaged interactive effect. As such, there is something unique about their experience which could be understood to focus clinical, public health and wider public policy. For example, compared to a general approach, interventions that differentially improve their access to healthcare services or obesity treatments may be more cost-effective and reduce health inequalities. Research that studies the biopsychosocial factors (e.g. weight stigma) that contribute to this intersectional effect may further improve these interventions. Without considering intersectionality it would be easy to exacerbate existing inequalities.

### Strengths and limitations

This study used a large biomedical database with multiple demographic and anthropomorphic measurements. This enabled a thorough analysis of multiple different axes of intersectionality within general and central obesity. The estimation of the complex intersectional structure through MAIHDA is reliable compared to fixed-effect approaches [14].

95% of UK Biobank participants are White ethnicity, similar to the 2001 UK census (94.5%), but greater than the 2021 census (81.7%) [49, 50]. As such, the UK biobank is mis-representative of the current ethnic makeup of the UK. While grouping by key ethnic categories was necessary due to statistical power, aggregating groups (e.g. Black African and Black Caribbean) into super-categories may obscure important within-group heterogeneity. These groups may differ in cultural practices, migration histories, and structural exposures that influence obesity risk. Similarly, whilst these ethnic groups may be heterogenous we still chose to group them as opposed to excluding a specific subgroup (e.g. Chinese). Excluding a subgroup would result in invisibility within the research which may reinforce disadvantage [51].

Multilevel models shrink random-effects estimates inversely to sample size. Under sampled social strata risk underestimation of their intersectional effect, risking type 2 error. In this study, this would be the case for Black males in low deprivation neighbourhoods. However, this is balanced against the risk of type 1 error if using a traditional single-level approach.

It is unclear at what geographic level neighbourhood deprivation takes effect. This study assumes a neighbourhood effect at the lower level super output area and data zones for England and Scotland.

Individual deprivation can be measured in multiple ways, such as socioeconomic class, education and income [52]. Income provided the best strata structure as well as being the most direct measure of material resource, so we felt it was the best measure. It was highly correlated with other measures of individual deprivation.

## Conclusion

When defined by BMI and WHtR, 11.1% and 17.3% of variation in general and central obesity is due to inequality between our strata. Of this, 19.5% and 18.2% is interactive as opposed to additive. In effect, this results in a 1.8 kg/m^2^ (26.9%) increase in the range of BMI compared to only using an additive approach. In general, both individual and neighbourhood deprivation are associated with higher obesity rates. However, the relationship between deprivation and obesity is heterogenous, with inverted relationships identified for some social groups.

In general, people of black ethnicity have the greatest probability of overweight or obesity (up to 87.42%) whilst white and Asian ethnicities have the lowest. However, in general, Black women experience a disadvantaged interactive effect and Black men experience an advantaged interactive effect, which act to widen and narrow the inequality gap respectively.

## Supplementary information

Full female waist to height ratio table (supplementary)

Full male waist to height ratio table (supplementary)

Full female BMI table (supplementary)

Full female FMI table (supplementary)

Full male BMI table (supplementary)

Full male FMI table (supplementary)

Supplementary material

## Data Availability

UK Biobank holds the data, so authors cannot release this. However, code (R 4.4.1) will be available on request.
