# Supplementary material for: Intersectional inequality in general and central obesity: cross-sectional UK Biobank study
Source: Int J Obes (Lond). 2026 Jan 5;50(4):731–40. doi: 10.1038/s41366-025-01984-2 (PMC13056582; doi:10.1038/s41366-025-01984-2)
Supplement: Supplementary file 7 — Supplementary material [file 41366_2025_1984_MOESM7_ESM.pdf]

Supplementary material

Missing Data Summary

Analysis of Missing Values by Variable

Variable Name	Number of Missing Values	Missing Percentage (%)
Index of Multiple Deprivation	12724	2.65
Fat Mass Index	10193	2.12
Income	5965	1.24
Body Mass Index	2982	0.62
Ethnicity	2595	0.54
Height	2431	0.51
Waist Circumference	2083	0.43
Sex	0	0.00
Age category	0	0.00

Table S1: Table of missing data by variable

Sample size of strata

Sample size	Number of strata
Less than 10	3
10-20	11
20-30	20
30-50	32
50-100	77
More than 100	177

Table S2: Number of strata by sample size

Sex	Income	Ethnicity	Age	Neighbourhood Deprivation	Strata N	Observed BMI	Predicted BMI (fixed effects)	Predicted BMI (fixed and random effects)	Random Effect - BMI. (95% CI)	Probability of Overweight or Obesity	Random Effect Obesity (95% CI)
Female											
Head											
Female	PNTS	Black	55 or above	High	263	31.33	29.93	31.43	4.79 (2.83, 6.76)	87.38	1.62 (1.17, 2.23)
Female	<£18,000	Black	55 or above	High	245	31.28	30.19	31.41	3.91 (1.75, 5.87)	87.32	1.42 (1.00, 2.04)
Female	<£18,000	Black	<55	High	484	30.63	29.77	30.92	3.80 (1.80, 5.75)	84.00	1.28 (1.00, 1.65)
Female	<£18,000	Black	55 or above	Moderate-High	141	30.45	29.68	30.54	2.80 (0.28, 5.37)	84.19	1.31 (0.92, 1.92)
Female	PNTS	Black	<55	High	308	30.28	29.53	30.51	3.17 (1.58, 4.86)	80.71	1.12 (0.85, 1.48)
Tail											
Female	>£52,000	White	<55	Low	14,018	25.06	26.12	25.41	-2.75 (-3.84, -1.66)	45.06	0.69 (0.59, 0.80)
Female	PNTS	Asian	<55	Low	123	24.83	25.91	25.39	-2.10 (-4.66, 0.48)	49.13	0.95 (0.65, 1.43)
Female	£31,000-£52000	Asian	<55	Low	130	24.69	25.69	25.20	-1.96 (-4.44, 0.65)	47.89	0.83 (0.57, 1.26)
Female	>£52,000	Asian	<55	Moderate-Low	163	24.53	25.74	25.06	-2.74 (-4.80, -0.69)	45.68	0.71 (0.48, 1.07)
Female	>£52,000	Asian	<55	Low	208	24.21	25.33	24.71	-2.46 (-4.74, -0.32)	39.32	0.84 (0.59, 1.18)
Male											
Head											
Male	<£18,000	Black	55 or above	Moderate-Low	22	29.27	29.61	29.66	0.19 (-3.30, 3.48)	85.31	0.98 (0.62, 1.62)
Male	£31,000-£52000	Black	55 or above	High	59	28.67	29.99	29.54	-1.75 (-5.03, 1.40)	87.42	0.99 (0.63, 1.61)
Male	>£52,000	Black	<55	High	85	29.32	29.16	29.46	1.04 (-1.89, 3.83)	84.45	1.30 (0.83, 2.02)
Male	<£18,000	Black	55 or above	Moderate-High	79	28.56	29.98	29.36	-2.19 (-5.11, 0.43)	86.96	0.93 (0.58, 1.46)
Male	£31,000-£52000	Black	55 or above	Moderate-High	51	28.8	29.48	29.33	-0.43 (-3.75, 2.59)	85.92	0.97 (0.59, 1.59)
Tail											
Male	£31,000-£52000	Asian	<55	Low	117	26.04	25.96	26.23	1.12 (-1.45, 3.67)	62.62	0.85 (0.53, 1.35)
Male	>£52,000	Asian	55 or above	Low	146	25.89	25.95	26.18	0.85 (-1.61, 3.06)	62.41	0.86 (0.58, 1.29)
Male	£18,000-£31000	Asian	<55	Low	46	25.92	26.01	26.13	0.45 (-2.72, 3.12)	60.80	1.16 (0.71, 1.96)
Male	£31,000-£52000	Asian	55 or above	Low	96	25.54	26.33	26.04	-1.02 (-3.66, 1.80)	63.08	0.73 (0.50, 1.11)
Male	>£52,000	Asian	<55	Low	229	25.67	25.6	25.92	1.22 (-0.94, 3.61)	58.09	1.00 (0.67, 1.50)

Table S3: Head and tail table for predicted BMI and obesity classifications. Strata are ranked based on predicted BMI (with RE). Strata were included if they were in the top or bottom 5 for predicted BMI (with RE) by male and female sex. The predicted outcome with only fixed and fixed and random effects, as well as the random effect are included. Fixed effects detail the additive effect and random effect the interactive. The random effect obesity is the random effect for the obesity v normal weight model, detailing the interactive effect for the respective strata. PNTS represents prefers not to say.

Sex	Income	Ethnicity	Age	Neighbourhood Deprivation	Strata N	Observed FMI	Predicted FMI (fixed effects)	Predicted FMI fixed and random effects)	Random Effect FMI (95% CI)	Probability of overweight or obesity	Random Effect Obesity (95% CI)
Female											
Head											
Female	<£18,000	Black	55 or above	High	245	12.95	12.83	13.58	5.80 (1.42, 9.85)	84.96	1.44 (1.09, 1.89)
Female	PNTS	Black	55 or above	High	263	12.88	12.66	13.50	6.45 (2.53, 10.92)	84.94	1.63 (1.20, 2.16)
Female	<£18,000	Black	<55	High	484	12.21	11.84	12.88	8.39 (5.08, 11.53)	77.38	1.35 (1.06, 1.70)
Female	<£18,000	Black	55 or above	Moderate-High	141	12.36	12.39	12.87	3.93 (-1.02, 8.81)	81.43	1.29 (0.95, 1.72)
Female	£18,000-£31000	Black	55 or above	High	136	12.18	12.48	12.82	2.63 (-2.43, 7.52)	80.43	1.17 (0.82, 1.62)
Tail											
Female	>£52,000	Mixed	<55	Low	169	8.36	9.13	9.01	-1.70 (-6.48, 2.83)	43.27	1.05 (0.74, 1.49)
Female	>£52,000	Asian	<55	Moderate-Low	163	8.23	9.34	8.99	-3.99 (-9.26, 1.27)	44.91	0.74 (0.51, 1.05)
Female	>£52,000	White	<55	Low	14,018	8.29	9.17	8.86	-3.34 (-5.47, -1.24)	40.08	0.82 (0.72, 0.93)
Female	£31,000-£52000	Asian	<55	Low	130	8.00	9.28	8.82	-4.80 (-9.74, 0.29)	43.84	0.76 (0.52, 1.11)
Female	>£52,000	Asian	<55	Low	208	7.87	9.03	8.59	-5.02 (-9.67, -0.70)	40.65	0.94 (0.69, 1.26)
Male											
Head											
Male	£31,000-£52000	Black	55 or above	High	59	7.58	8.51	8.33	-1.92 (-7.64, 3.88)	80.44	0.83 (0.58, 1.26)
Male	<£18,000	Black	55 or above	Moderate-High	79	7.52	8.48	8.27	-2.57 (-8.13, 2.74)	82.19	0.96 (0.63, 1.42)
Male	<£18,000	Black	55 or above	Moderate-Low	22	7.59	8.26	8.24	-0.60 (-7.53, 6.35)	79.97	0.99 (0.64, 1.54)
Male	<£18,000	White	55 or above	High	8,187	7.63	7.93	8.16	2.85 (0.91, 4.85)	77.01	1.20 (1.05, 1.37)
Male	<£18,000	Black	55 or above	High	204	7.39	8.78	8.14	-7.46 (-11.60, -3.25)	80.53	0.63 (0.45, 0.88)
Tail											
Male	>£52,000	Mixed	<55	Moderate-High	68	5.83	6.64	6.46	-3.03 (-9.54, 2.82)	54.91	0.90 (0.60, 1.37)
Male	>£52,000	Asian	<55	Low	229	6.07	6.18	6.40	3.62 (-0.73, 8.01)	51.44	1.09 (0.78, 1.53)
Male	£31,000-£52000	Asian	<55	Low	117	5.96	6.35	6.37	0.19 (-4.82, 5.29)	54.29	0.76 (0.52, 1.12)
Male	£18,000-£31000	Asian	<55	Low	46	5.86	6.37	6.34	-0.47 (-6.62, 5.74)	55.09	1.03 (0.66, 1.63)
Male	£18,000-£31000	Mixed	<55	Low	28	5.35	6.45	6.27	-2.97 (-9.74, 3.76)	53.84	1.01 (0.64, 1.61)

Table S4: Head and tail table of the FMI and its associated obesity classification. Strata are ranked based on predicted FMI (with RE) . Strata were included if they were in the top or bottom 5 for predicted FMI by male and female sex The predicted outcome with only fixed and fixed and random effects, as well as the random effect are included. Fixed effects detail the additive effect and random effect the interactive. The random effect obesity is the random effect for the obesity v normal weight model, detailing the interactive effect for the respective strata. PNTS represents prefers not to say.

Sex	Income	Ethnicity	Age	Neighbourhood Deprivation	Strata N	Observed WHtR	Predicted WHtR (fixed effects)	Predicted WHtR (fixed and random effects)	Random Effect WHtR (95% CI)	Probability of increased or high	Random Effect Central Obesity (95% CI)
Female											
Head											
Female	<£18,000	Black	55 or above	High	245	0.59	0.57	0.59	3.29 (1.62, 4.81)	86.20	1.41 (0.99, 1.98)
Female	PNTS	Asian	55 or above	High	160	0.59	0.57	0.59	3.61 (1.57, 5.66)	85.95	1.74 (1.15, 2.67)
Female	<£18,000	Mixed	55 or above	High	146	0.57	0.57	0.58	1.75 (-0.39, 4.02)	81.82	1.18 (0.76, 1.77)
Female	<£18,000	Asian	55 or above	High	160	0.58	0.57	0.58	1.39 (-0.53, 3.15)	85.35	1.13 (0.75, 1.74)
Female	<£18,000	Black	55 or above	Moderate-High	141	0.58	0.57	0.58	2.13 (0.15, 3.96)	83.39	1.21 (0.78, 1.86)
Tail											
Female	£31,000-£52000	White	<55	Low	8,009	0.49	0.50	0.49	-1.28 (-2.16, -0.39)	40.31	1.01 (0.85, 1.21)
Female	>£52,000	Asian	<55	Low	208	0.49	0.50	0.49	-2.16 (-3.98, -0.25)	43.46	0.85 (0.57, 1.32)
Female	>£52,000	Mixed	<55	Low	169	0.49	0.50	0.49	-1.04 (-3.20, 0.91)	37.52	1.10 (0.72, 1.68)
Female	>£52,000	White	<55	Moderate-Low	8,719	0.49	0.50	0.49	-1.75 (-2.61, -0.91)	38.05	0.86 (0.73, 1.01)
Female	>£52,000	White	<55	Low	14,018	0.48	0.49	0.49	-1.62 (-2.46, -0.73)	34.19	0.91 (0.77, 1.08)
Male											
Head											
Male	<£18,000	Asian	55 or above	High	236	0.58	0.59	0.59	-0.81 (-2.51, 0.78)	92.75	1.17 (0.78, 1.75)
Male	£18,000-£31000	Asian	55 or above	Moderate-Low	118	0.58	0.57	0.58	1.81 (-0.15, 3.85)	89.82	1.53 (0.96, 2.43)
Male	<£18,000	White	55 or above	High	8,187	0.58	0.58	0.58	0.33 (-0.52, 1.09)	87.35	1.01 (0.86, 1.19)
Male	<£18,000	Mixed	55 or above	Moderate-Low	37	0.58	0.57	0.58	0.93 (-1.75, 3.82)	86.92	1.11 (0.62, 1.96)
Male	<£18,000	Mixed	55 or above	Moderate-High	76	0.57	0.58	0.58	-0.32 (-2.85, 2.02)	87.89	0.94 (0.55, 1.52)
Tail											
Male	>£52,000	Mixed	<55	Low	134	0.53	0.52	0.53	2.97 (0.99, 5.03)	69.47	1.78 (1.15, 2.83)
Male	>£52,000	White	<55	Moderate-Low	8,096	0.53	0.52	0.53	3.27 (2.39, 4.17)	70.32	1.52 (1.29, 1.78)
Male	>£52,000	White	<55	Low	12,599	0.53	0.51	0.53	3.79 (2.85, 4.65)	68.59	1.65 (1.42, 1.94)
Male	>£52,000	Black	<55	Low	55	0.51	0.53	0.52	-0.70 (-3.19, 2.01)	65.45	0.75 (0.44, 1.25)
Male	>£52,000	Asian	<55	Low	229	0.52	0.52	0.52	0.45 (-1.24, 2.12)	67.32	0.71 (0.48, 1.05)

Table S5: Head and tail table of waist to height ratio and its associated obesity classification. Strata are ranked based on predicted waist to height ratio (with RE) . Strata were included if they were in the top or bottom 5 for predicted waist to height ratio by male and female sex. The predicted outcome with only fixed and fixed and random effects, as well as the random effect are included. Fixed effects detail the additive effect and random effect the interactive. The probability of increased or high is relative to normal waist to height ratio. The random effect of central obesity is the random effect for the high v normal model, detailing the interactive effect for the respective strata. PNTS represents prefers not to say.

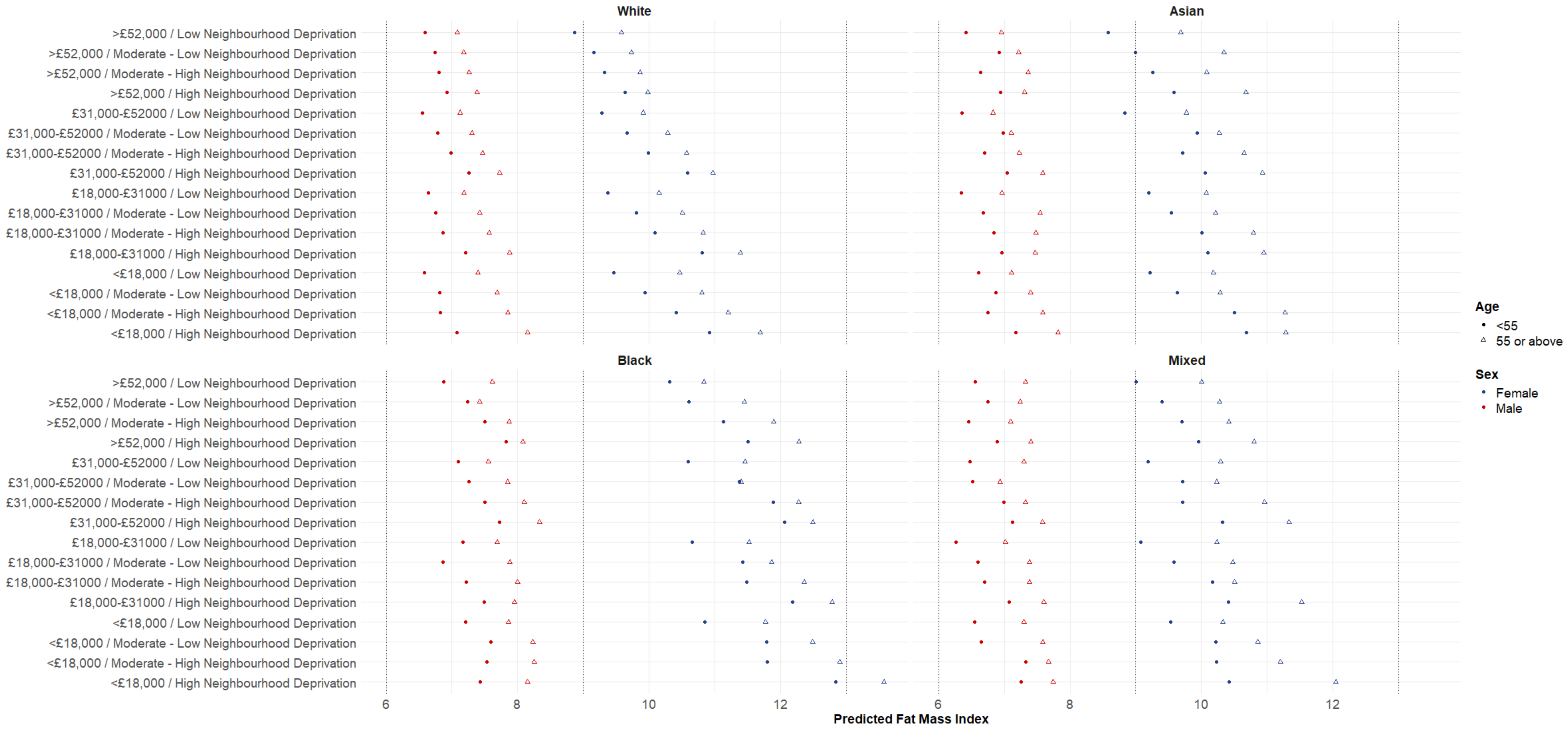

Figure S1: *Predicted strata fat mass index by ethnicity groupings. Predictions are made used fixed and random effect, so represent both additive and interactive effects. Household income and neighbourhood deprivation groupings are detailed on the y axis. Male observations are in red, with female observations in blue. Aged <55 is plotted with a circle, aged 55 or above is a triangle. Dotted vertical lines indicate the overweight (Female - FMI 9kg/m^2, Male – FMI 6kg/m^2) and obesity (Female - FMI 13kg/m^2, Male – FMI 9kg/m^2) classifications.*

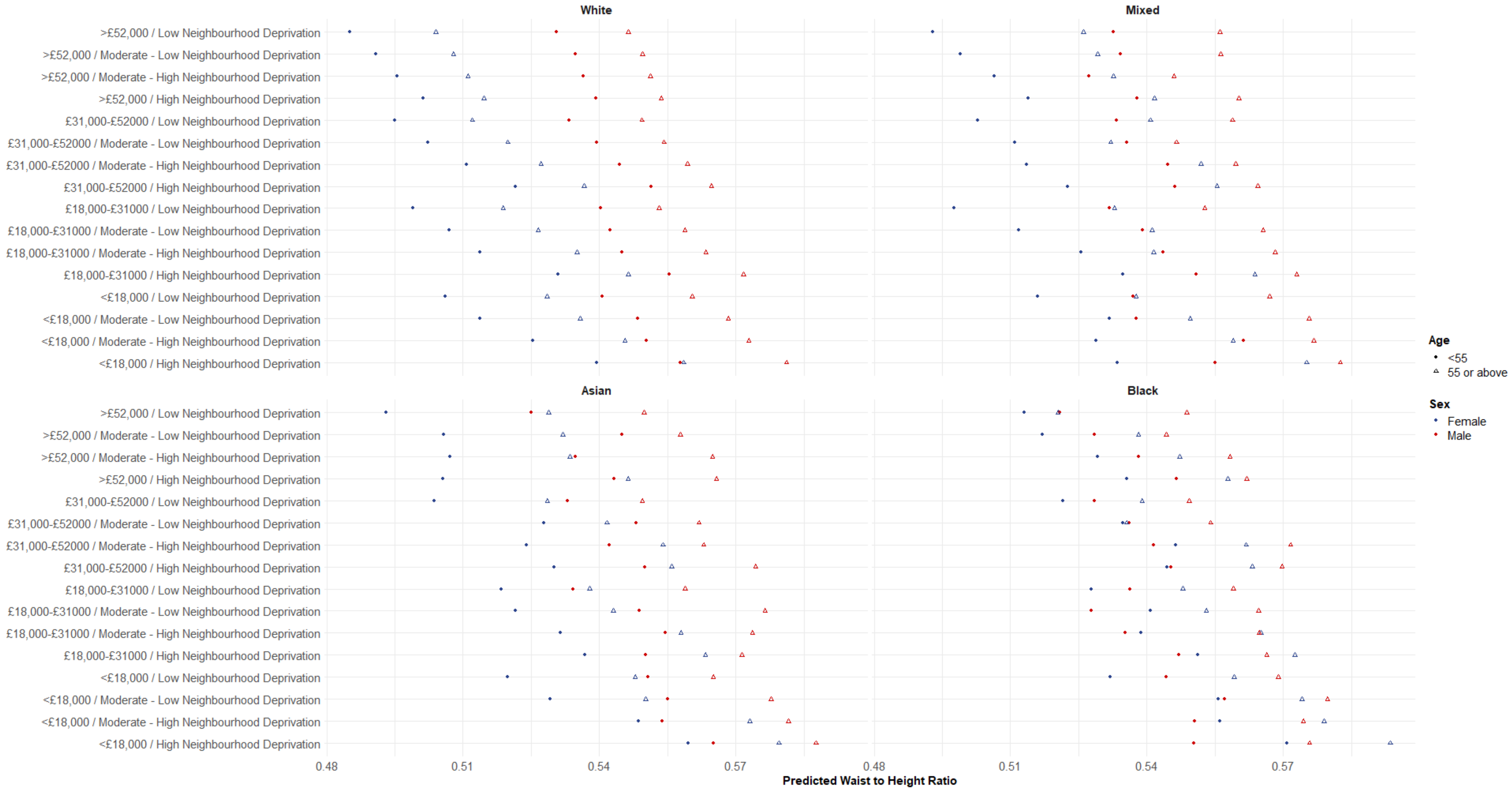

Figure S2: *Predicted strata waist to height ratio by ethnicity groupings. Predictions are made used fixed and random effect, so represent both additive and interactive effects. Household income and neighbourhood deprivation groupings are detailed on the y axis. Male observations are in red, with female observations in blue. Aged <55 is plotted with a circle, aged 55 or above is a triangle*

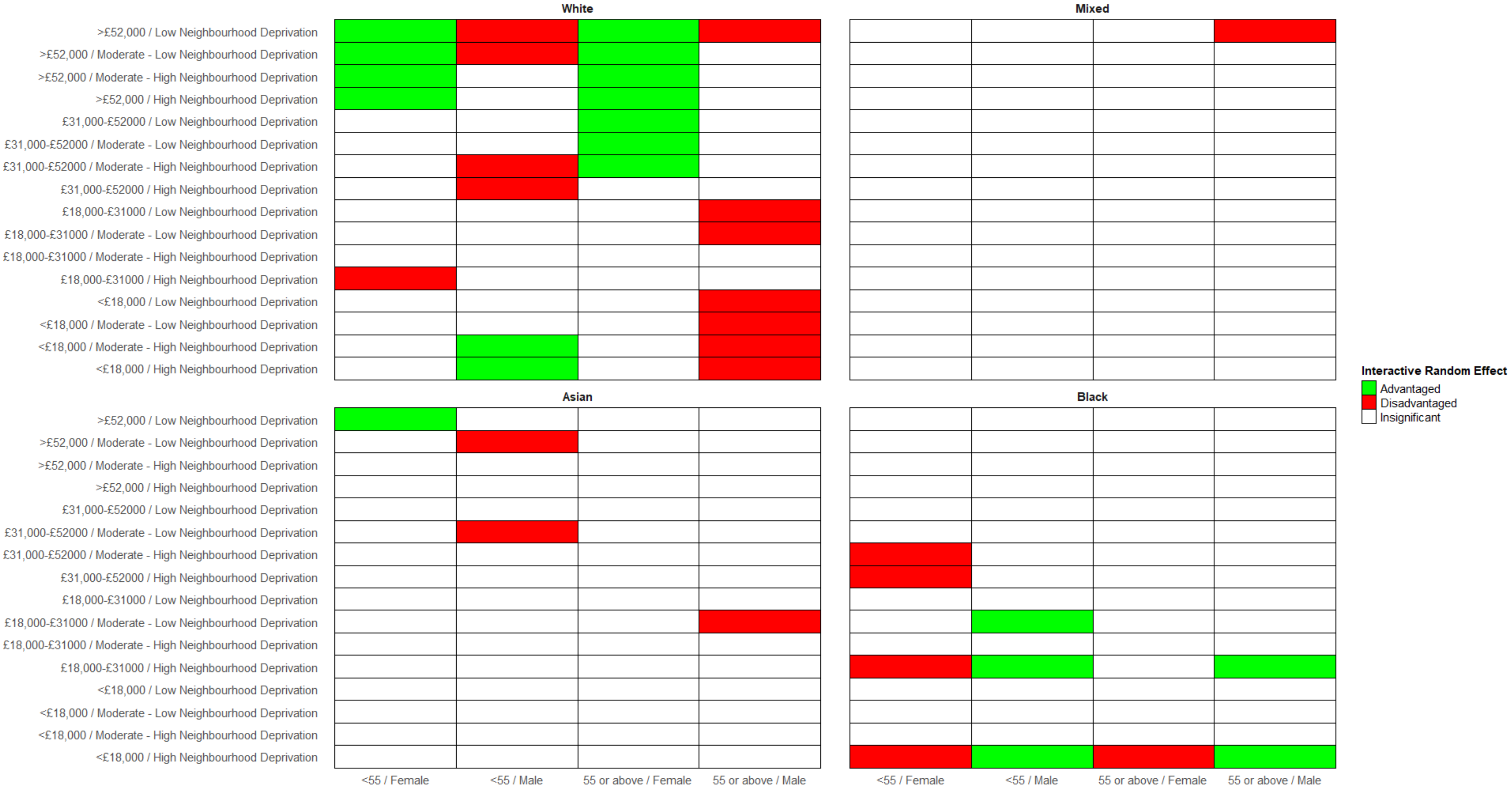

Figure S3: *Heatmap of interactive random effect per strata for fat mass index, by ethnicity grouping. Statistically significant disadvantaged interactive effects are red, statistically significant advantaged interactive effects are green. Statistically insignificant interactive effects are blank.*

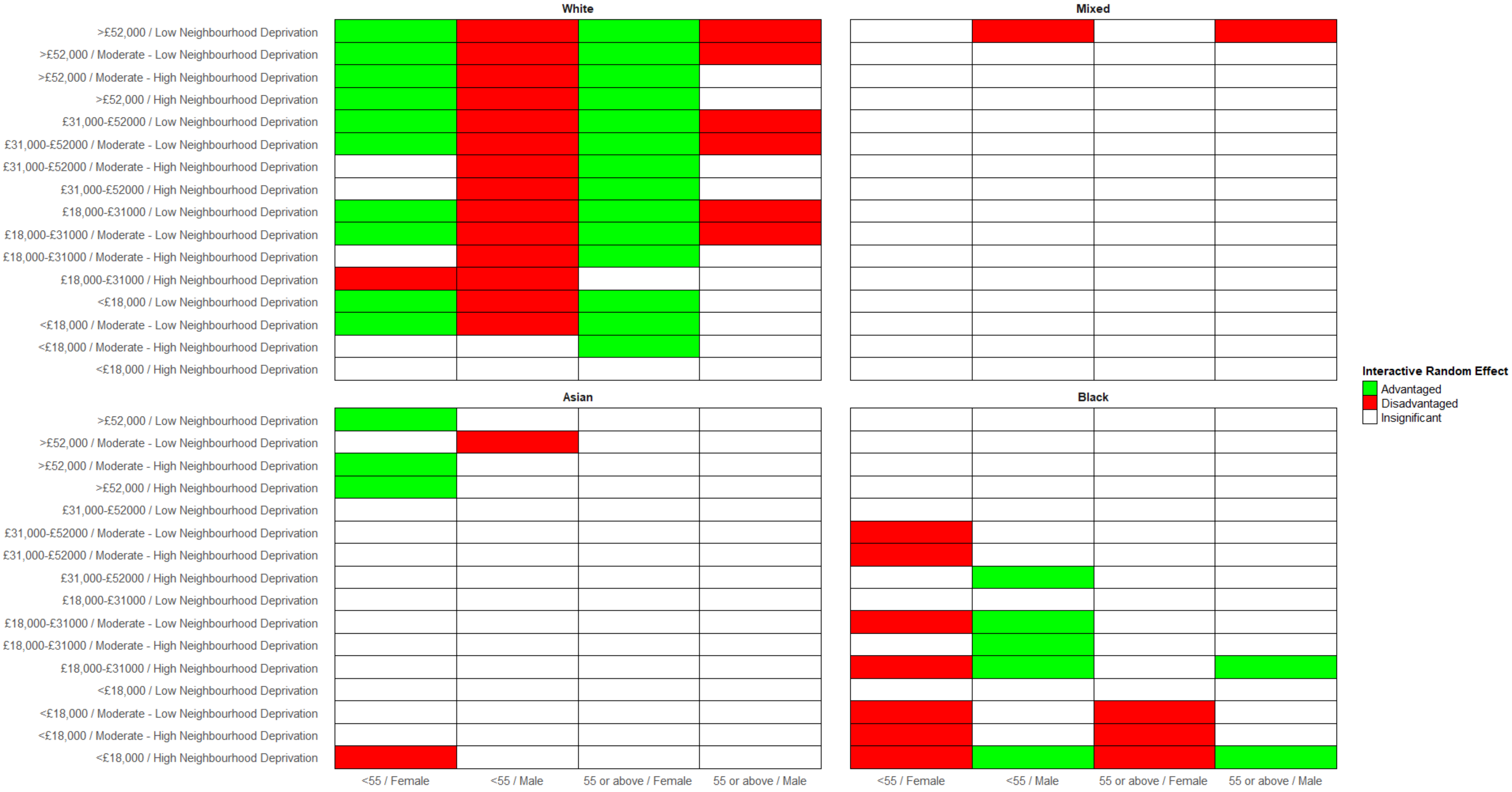

Figure S4: *Heatmap of interactive random effect per strata for waist to height ratio, by ethnicity grouping. Statistically significant disadvantaged interactive effects are red, statistically significant advantaged interactive effects are green. Statistically insignificant interactive effects are blank*
